# Exploring the potential of selective FKBP51 inhibitors on melanoma: an investigation of their in vitro and in vivo effects

**DOI:** 10.1038/s41420-025-02430-y

**Published:** 2025-04-03

**Authors:** Laura Marrone, Valeria Di Giacomo, Chiara Malasomma, Marialuisa Alessandra Vecchione, Felix Hausch, Massimiliano Cacace, Lucia D’Esposito, Martina Tufano, Paolo D’Arrigo, Maria Fiammetta Romano, Simona Romano

**Affiliations:** 1 Department of Molecular Medicine and Medical Biotechnologies, Federico II University, Naples, Italy; 2 Technical University Darmstadt Institute of Organic Chemistry and Biochemistry, Darmstadt, Germany; 3 Centro Servizi Veterinari, Federico II University, Naples, Italy; 4 Samuel Oschin Comprehensive Cancer Institute, Cedars-Sinai Medical Center, Los Angeles, CA USA

**Keywords:** Melanoma, Immunosurveillance

## Abstract

FKBP51 is a marker of melanocyte malignancy, correlating with vertical growth phase and lesion thickness. It promotes the typical features of epithelial to mesenchymal transition and sustains apoptosis resistance. The present study aimed to assess in vitro and in vivo the efficacy against melanoma of selective small molecules targeting FKBP51, called SAFits. Our findings reveal differing outcomes for SAFits in vitro compared to in vivo. SAFit increased the doxorubicin and dacarbazine cytotoxicity of cultured melanoma cells and was effective in impairing NF-κB activity and related pro-survival genes. Moreover, SAFit affected TGF-β-signaling and reduced the capability of melanoma cells to migrate through transwell filters and invade the matrigel. Unexpectedly, SAFit was ineffective in reducing tumor growth in a syngeneic melanoma mouse model. A study of the tumor microenvironment revealed an enrichment of M2 macrophages in SAFit-treated mice. Western blot assay showed reduced levels of perforin in protein extracted from SAFit-treated tumor samples. Ex-vivo experiments showed that M1 and M2 macrophages exerted an opposite effect on the cytotoxic capacity of CD8 T cells, supporting the hypothesis that enrichment in M2 macrophages induced by SAFit could accelerate the exhaustion of CD8 lymphocytes. In conclusion, our study shows that selective FKBP51 targeting agents hinder the intrinsic pro-survival pathways of melanoma cells but simultaneously exacerbate immune suppression within the tumor microenvironment, and, therefore, they have not proven to be effective in vivo to counteract melanoma growth.

## Introduction

FKBP51 binding protein (FKBP51) is an immunophilin and co-chaperone encoded by the *FKBP5* gene [1]. Structurally, the protein features a C-terminal tetratricopeptide repeat (TPR) domain that facilitates protein-protein interactions, and an N-terminal region that contains a binding domain for immunosuppressant agents such as rapamycin [2]. This binding domain possesses enzymatic activity, specifically peptidyl-prolyl isomerase (PPIase), which is inhibited by the binding of drug ligands like FK506 and rapamycin [2]. Due to its unique structure, FKBP51 can act both as a scaffold and an enzyme, playing significant roles in various biological processes within the cell [3]. FKBP51 takes part to NF-κB signalosome, allowing a correct assembly and efficient kinase function of the IKK kinase complex [4]. This immunophilin is highly expressed in immune cells, where it was first cloned [1], and it plays an important role in activating the immune response [5]. FKBP51 is aberrantly expressed by melanoma tumors [6]. A wide number of studies highlight the pivotal role exerted by FKBP51 in melanoma progression [7] and therapy-resistance [4, 6, 8, 9]. FKBP51 promotes the recruitment of pSmad to p300 coactivator, increasing the sensitivity of melanoma cells to the pro-tumoral TGF-β effects [7], particularly it sustains the typical features of epithelial to mesenchymal transition (EMT) [10].

In the last years, novel small molecules targeting FKBP51 have been generated [11]. These small molecules are called “SAFit” for their selective antagonism of FKBP51 by induced fit [11, 12]. SAFits show a huge affinity for FKBP51 (Ki = 4 and 6 nM for SAFit 1 and SAFit 2, respectively) but a very poor affinity for the similar FKBP52 (Ki ≥ 50.000 nM) [11].

This study aimed to investigate the anti-melanoma effects of SAFit, both in vitro and in vivo. Our results indicate that while SAFit effectively inhibited pro-tumor signaling pathways in melanoma cell cultures, it failed to restrain tumor growth in a syngeneic melanoma mouse model. This discrepancy was attributed to the accelerated development of an immunosuppressive microenvironment.

## Results

### Selective inhibitors of FKBP51 enhance doxorubicin-induced cell death of melanoma cells

**Fig. 1:**
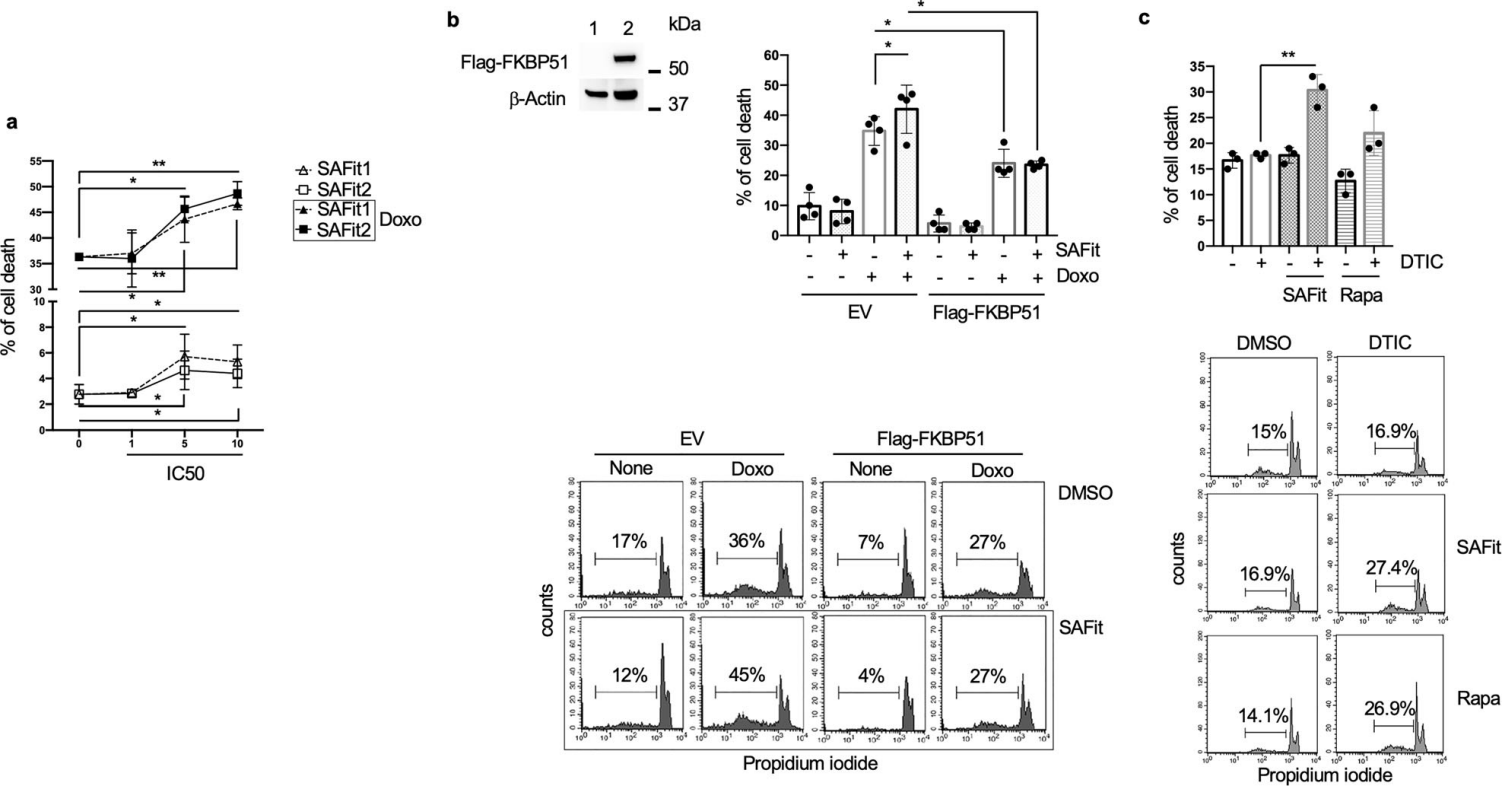
SAFit enhances melanoma cell death. **a** Dose response assay of SAFit 1 and 2 on Doxo-induced cell death. A375 melanoma cells were cultured in the presence or absence of 3 μM Doxo and/or SAFit 1 (4, 20 and 40 nM) and SAFit 2 (6, 30 and 60 nM). After a 48 h incubation, cell death was assessed by measuring the proportion of hypodiploid cells by flow cytometry. **b** (Left) Western blot assay showing exogenous levels of FKBP51, β-Actin was used as loading control. Full length western blots are shown as Supplemental Material. (Right) Graphical representation of cell death values (*N* = 4) from cultures of Flag-FKBP51 and EV incubated with 3 μM Doxo and/or 20 nM SAFit 1. After a 48 h incubation, cells were harvested and analysed by PI incorporation and flow cytometry. Representative flow cytometry histograms of PI incorporation are shown below. **c** SAFit improves dacarbazine (DTIC)-induced cell death. SAN melanoma cells were cultured in the presence or absence of 27 μM DTIC and with or without 100 nM SAFit 1 or 100 nM rapamycin. After 48 h incubation cells were harvested and analysed by flow cytometry. The graph shows cell death values obtained from independent experiments (*N* = 3). Representative flow cytometry histograms of PI incorporation are shown below.

To address whether SAFits can induce melanoma cell death, we used A375 melanoma cell line and performed a dose response assay of SAFits at doses correspondent to 1, 5 and 10x of the IC50 (SAFit 1: 4, 20 and 40 nM; SAFit 2: 6, 30 and 60 nM) [11, 12]. In addition, as a previous study showed that FKBP51-inhibition by rapamycin sensitized melanoma cells to doxorubicin-induced apoptosis [8], cells were also incubated for 48 h, in the presence, or not, of 3 μM doxorubicin (Doxo). Cell death was measured by PI incorporation and flow cytometry. SAFits alone produced only a slight increase of cell death at the doses 5 and 10x IC50 (Fig. 1a, lower). Melanoma cells cultured with doxorubicin died as an average by 36.3 ± 0.6% (Fig. 1a). Such percentage was increased to 43.6 ± 4.5% (p*) and 45.6 ± 2.3% (p*) with 20 nM SAFit 1 and 30 nM SAFit 2, respectively (*N* = 3); and to 46.6 ± 1.1% (p**) and 48.6 ± 2.3%, (p**) with 40 nM SAFit 1 and 60 nM SAFit 2, respectively (*N* = 3). To confirm that SAFit-induced chemo-sensitization was due to FKBP51 inhibition, we used SAFit 1 and investigated its effect on FKBP51-over expressing melanoma cells (Flag-FKBP51) (Fig. 1b). Cells transfected with the empty vector served as control (EV) (Fig. 1b). Cell death of Flag-FKBP51was reduced in comparison with that of EV, in both Doxo (p*) and SAFit Doxo cultures (p*) (Fig. 1b, right). Moreover, the Doxo sensitizing effect of SAFit, which was observed in EV cultures (p*), was null with Flag-FKBP51 (Fig. 1b, right). The sensitizing effect of SAFit 1 was evaluated in combination with dacarbazine (DTIC), which is the standard chemotherapeutic agent used against melanoma. The low levels of cell death induced by DTIC were significantly increased by SAFit 1 (p**) but not rapamycin (Fig. 1c).

### FKBP51 inhibition by SAFit impairs doxorubicin induced NF-κB activation in melanoma cells

**Fig. 2:**
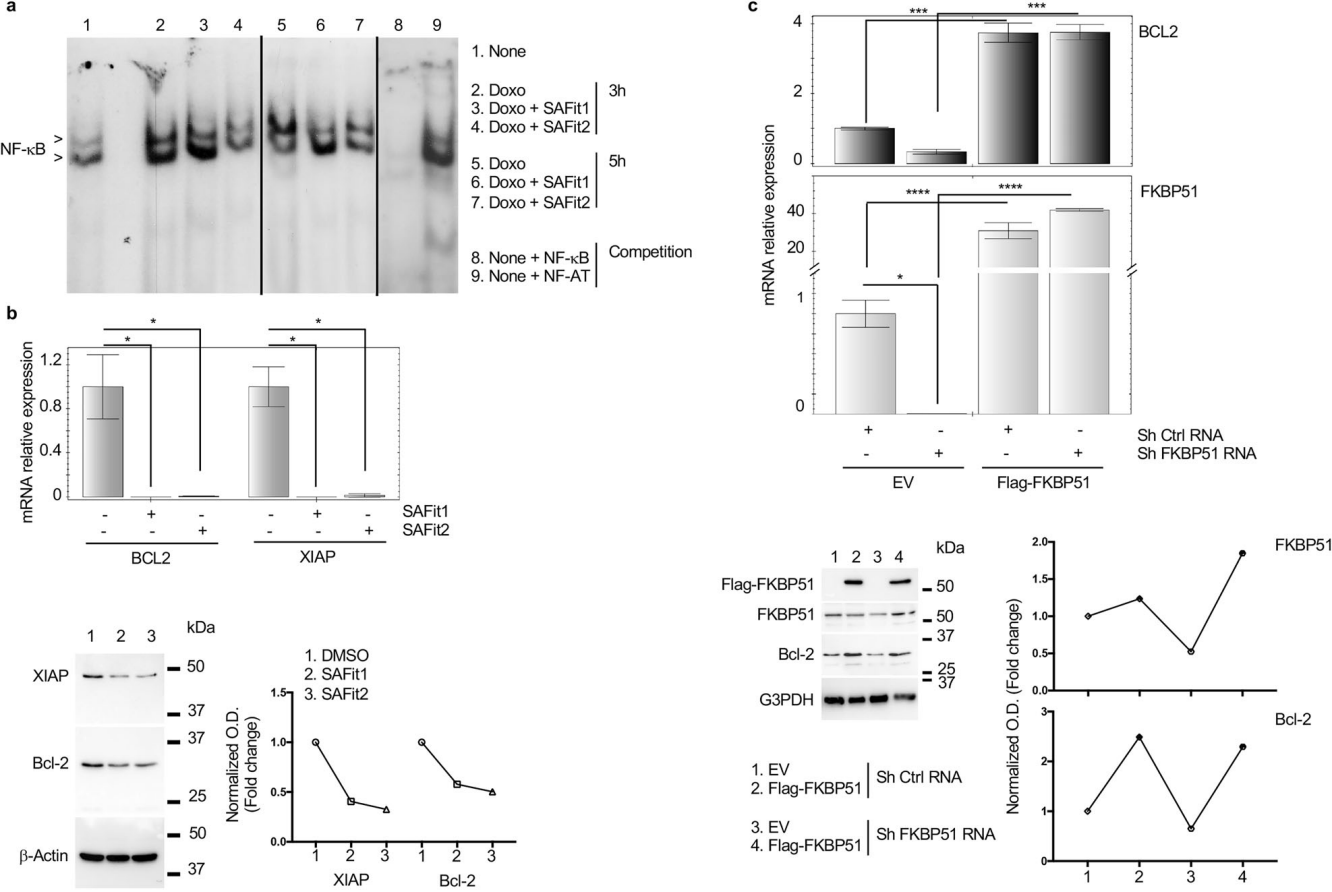
SAFits counteract NF-κB/Rel activation. **a** Electrophoretic mobility shift assay (EMSA) of nuclear extracts obtained from SAN melanoma cells cultured with 3 μM Doxo and/or SAFit1 20 nM or SAFit2 30 nM, for 3 and 5 h. A competition assay with the same cold oligo or an unrelated (NF-AT) oligo suggests the specificity of NF-κB bands. Full gels are shown as Supplemental Material. **b** Relative normalized expression values of *BCL-2* and *XIAP* mRNA levels in SAN melanoma cells incubated for 24 h in the presence or absence of 20 nM SAFit1 or 30 nM SAFit2. Relative quantitation of the transcript was performed using co-amplified β-Actin as an internal control for normalization. (Lower), Western blot of protein extracted from the same cells for Bcl-2 and XIAP assay. Full gels are shown as Supplemental Material. **c** *BCL-2* and *FKBP51* mRNA levels in FKBP51-knocked down A375 melanoma cells (Sh FKBP51 RNA), transfected or not with Flag-FKBP51. (Lower), Western blot of protein extracted from the same cells for Bcl-2, FKBP51 and FLAG-FKBP51 assay. Full gels are shown as Supplemental Material.

Because doxorubicin-induced NF-κB activation is counteracted by FKBP51 inhibition [8, 13], we performed an electrophoretic mobility shift assay (EMSA) of nuclear extracts from melanoma cells to investigate the SAFit effect on NF-κB. Cells were treated with 3 μM doxorubicin for 3 and 5 h, in the presence or not of 20 nM SAFit 1 or 30 nM SAFit 2. As expected, NF-κB nuclear translocation was activated by Doxo and this effect resulted impaired in the presence of both SAFits (Fig. 2a). The two NF-κB bands, indicated by the arrows (Fig. 2a) corresponded to p65/p50 heterodimer (upper) and p50/p50 homodimer (lower) [8]. Doxorubicin appeared to increase both dimers at 3 h (lane 2) and especially p65/p50 heterodimer at 5 h (lane 5), compared to untreated (lane 1) (Fig. 2a). The decreasing effect of SAFit 2 (lane 4) was earlier than that of SAFit 1 (lane 3), this latter reduced the p65/p50 heterodimer at 5 h (lane 6) (Fig. 2a). Competition assay (lanes 8, 9) showed the NF-κB bands disappeared because of the cold NF-κB probe competition (lane 8) (Fig. 2a). Differently, the binding of nuclear extracts to the NF-κB probe was not competed by the unrelated NFAT cold oligo (lane 9) (Fig. 2a). In accordance with SAFit-induced NF-κB inhibition, the mRNA and protein levels of *BCL-2* and *XIAP*, which are anti-apoptotic genes under the direct NF-κB transcriptional control [14] (Fig. 2b), were reduced by SAFit. Using a cell line stably knocked down for FKBP51, namely ShFKBP51.2 [4], we confirmed the dependence on FKBP51 of melanoma *BCL-2* expression levels (Fig. 2c). Such levels resulted impaired in Sh FKBP51 RNA but not control cells (Sh Ctrl RNA); exogenous FKBP51 restored *BCL-2* expression levels in silenced melanoma cells (Fig. 2c).

#### FKBP51-dependent TGF-β pro-tumoral function is inhibited by SAFit in vitro

**Fig. 3:**
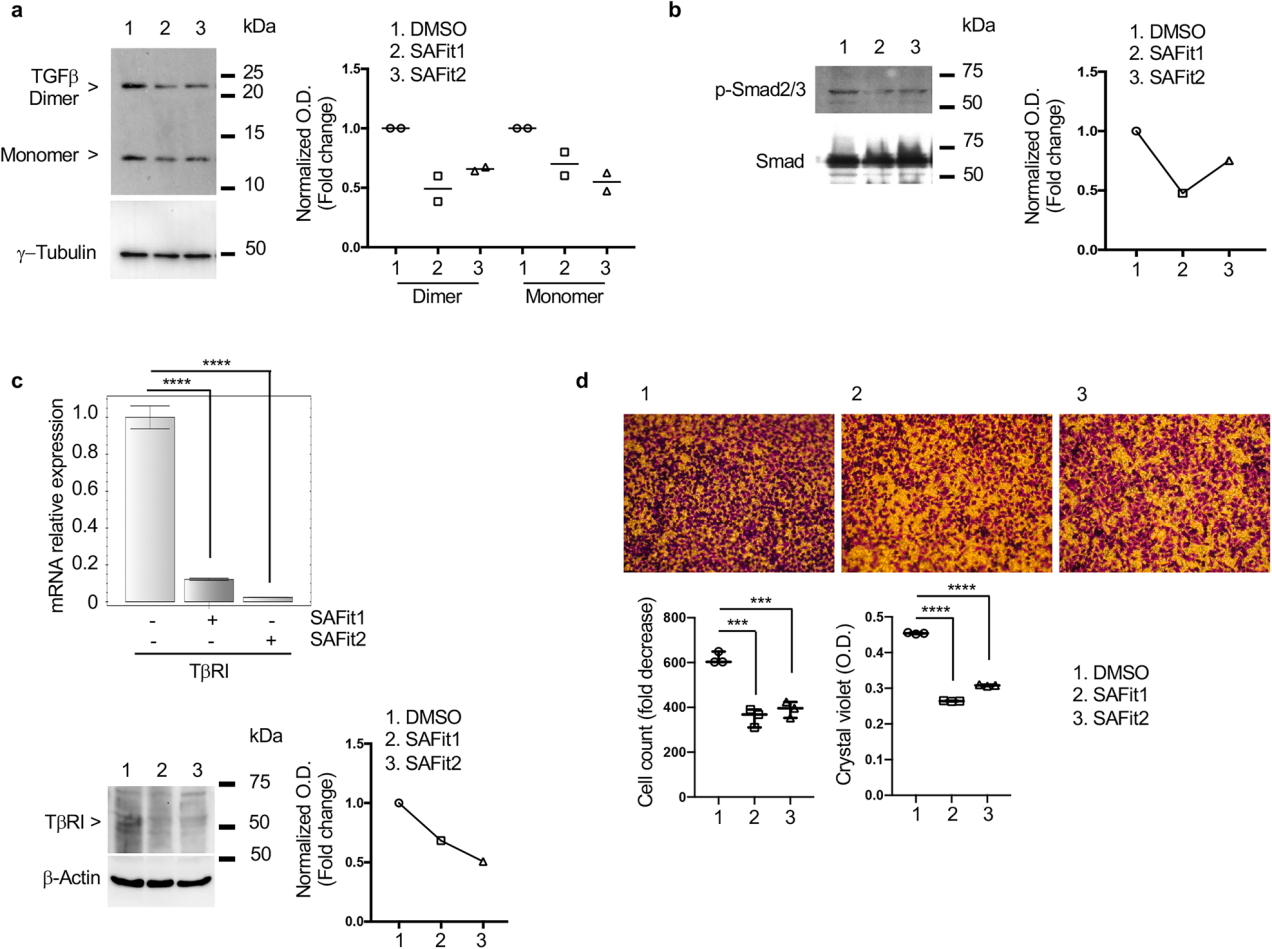
SAFits impair TGF-β expression and signaling. Western blot assay showing both the monomeric and dimeric form of TGF-β (**a**) and p-Smad2/3 (**b**) in A375 melanoma incubated in the absence or presence of 20 nM SAFit 1 or 30 nM SAFit 2. γ-Tubulin and total Smad were used as loading control. Full length western blots are shown as Supplemental Material. A densitometric analysis of bands was performed using ImageJ 1.42q for Macintosh. **c** Relative normalized expression values of *TβRI* mRNA levels in A375 melanoma cells incubated for 5 h in the presence or absence of 20 nM SAFit 1 or 30 nM SAFit2. Relative quantitation of the transcript was performed using co-amplified β-Actin as an internal control for normalization. Lower, Western blot of protein extracted from the same cells for TβRI assay. Full gels are shown as Supplemental Material. **d** Representative images (10x magnification fields containing ≥100 cells) of transwell migration and invasion assay of A375 melanoma cells cultured 48 h in the presence of 20 nM SAFit 1 or 30 nM SAFit2. Means of cell count values and crystal violet O.D. quantization obtained from different experiments (*N* = 3) are indicated below.

FKBP51 enhances the TGF-β pro-metastatic function in melanoma [10] and increases TGF-β expression at both mRNA and protein level [7]. A375 melanoma cells were treated, or not, with the selective inhibitors for 5 h and then whole lysates were analysed by Western blot. We found that TGF-β levels, both the dimeric and monomeric form, were decreased in the presence of SAFit (lanes 2 and 3) compared with untreated cells (lane 1) (Fig. 3a). The reduced TGF-β production determined a decrease in phosphorylated (p)-Smad2,3 levels (Fig. 3b). The transcript and protein levels of the receptor type I of TGF-β (TβRI), which is a direct transcriptional target of the cytokine, were reduced by the FKBP51 specific inhibitors (Fig. 3c). The ability of melanoma cells to migrate through transwell filters and invade matrigel was significantly hampered by SAFit (Fig. 3d).

#### SAFit does not impact melanoma growth in vivo

**Fig. 4:**
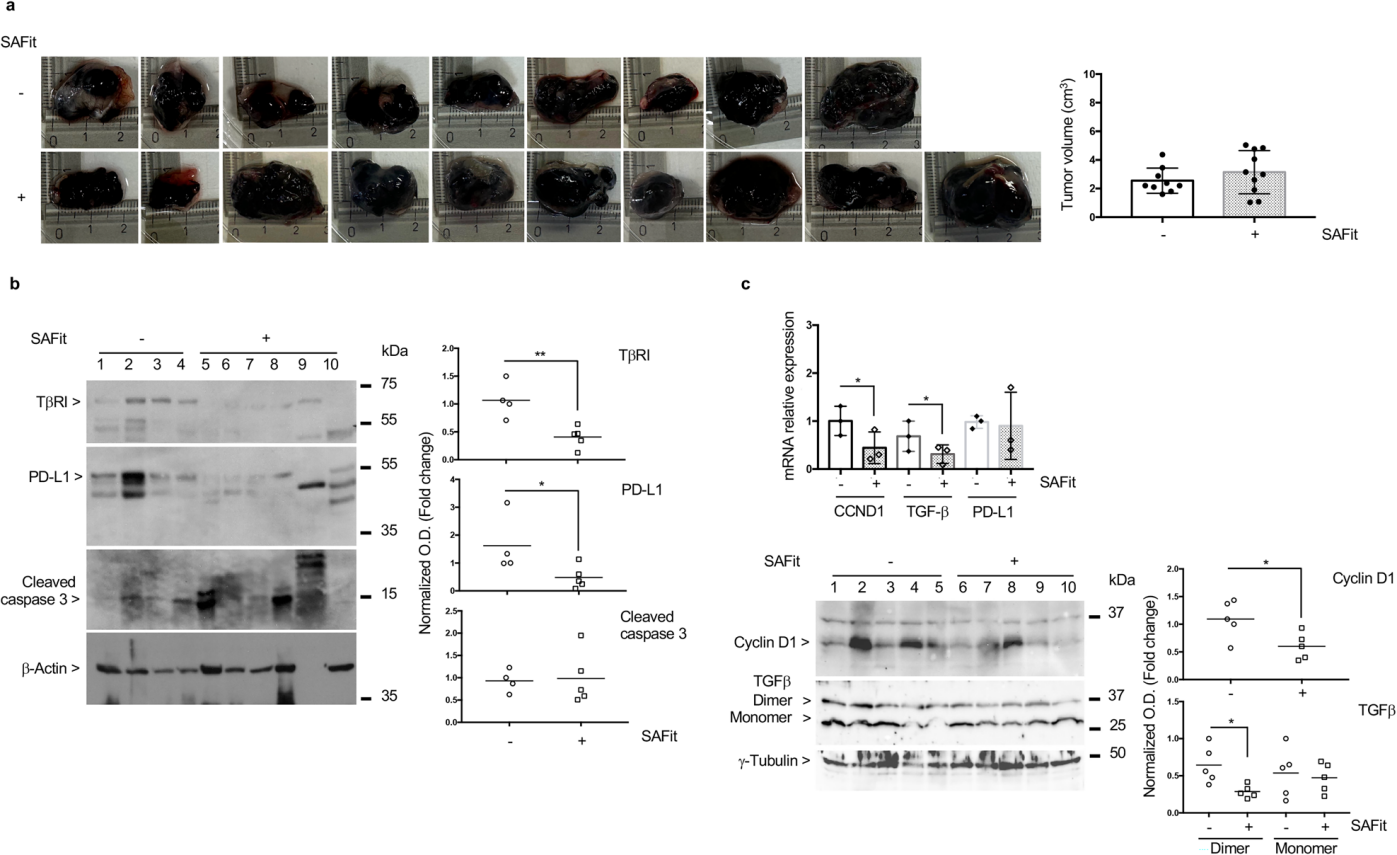
SAFit fails to restrain melanoma growth in vivo. **a** Tumors excised were photographed and measured. The graph on the right represents the volume measures. **b** Western blot assay of TβR1, PD-L1 and active Caspase 3 (analyzed on a blot run in parallel) expression levels in SAFit 2 -untreated and -treated tumors. β-Actin (analyzed on a blot run in parallel) was used as loading control. Full length western blots are shown as Supplemental Material. A densitometric analysis of bands was performed using ImageJ 1.42q for Macintosh. **c** Relative normalized expression values of *CCND1*, *TGFβ* and *PDL1* mRNA levels in SAFit-treated or untreated tumors. Lower, Western blot of protein extracted from the tumors for cyclin D and TGF-β assay. Full gels are shown as Supplemental Material.

Given the brilliant results obtained by in vitro treating melanoma cells with SAFits, we used SAFit 2 and investigated its effect on a syngeneic melanoma mouse model obtained with flank subcutaneous implantation of B16F10 melanoma cells. Unexpectedly, the pharmacological agent was ineffective in reducing tumor growth (Fig. 4a). Western blot assay of total lysates showed a decrease of TβR1 and PD-L1 in SAFit-treated tumors, while active caspase 3 was almost variable in treated and untreated tumors (Fig. 4b). Moreover, despite the tumor volumes appeared to be unaffected by SAFit, the transcript levels of *CCND1* and *TGF-β* were decreased in SAFit treated-tumors (Fig. 4c). A semiquantitative measure of the protein showed reduced cyclin D1 level in SAFit-treated tumors (Fig. 4c lower). Regarding TGF-β, we measured a slight but significant decrease in the dimer while no difference in the monomer (Fig. 4c lower).

#### SAFit induces M1 to M2 switch in TME

**Fig. 5:**
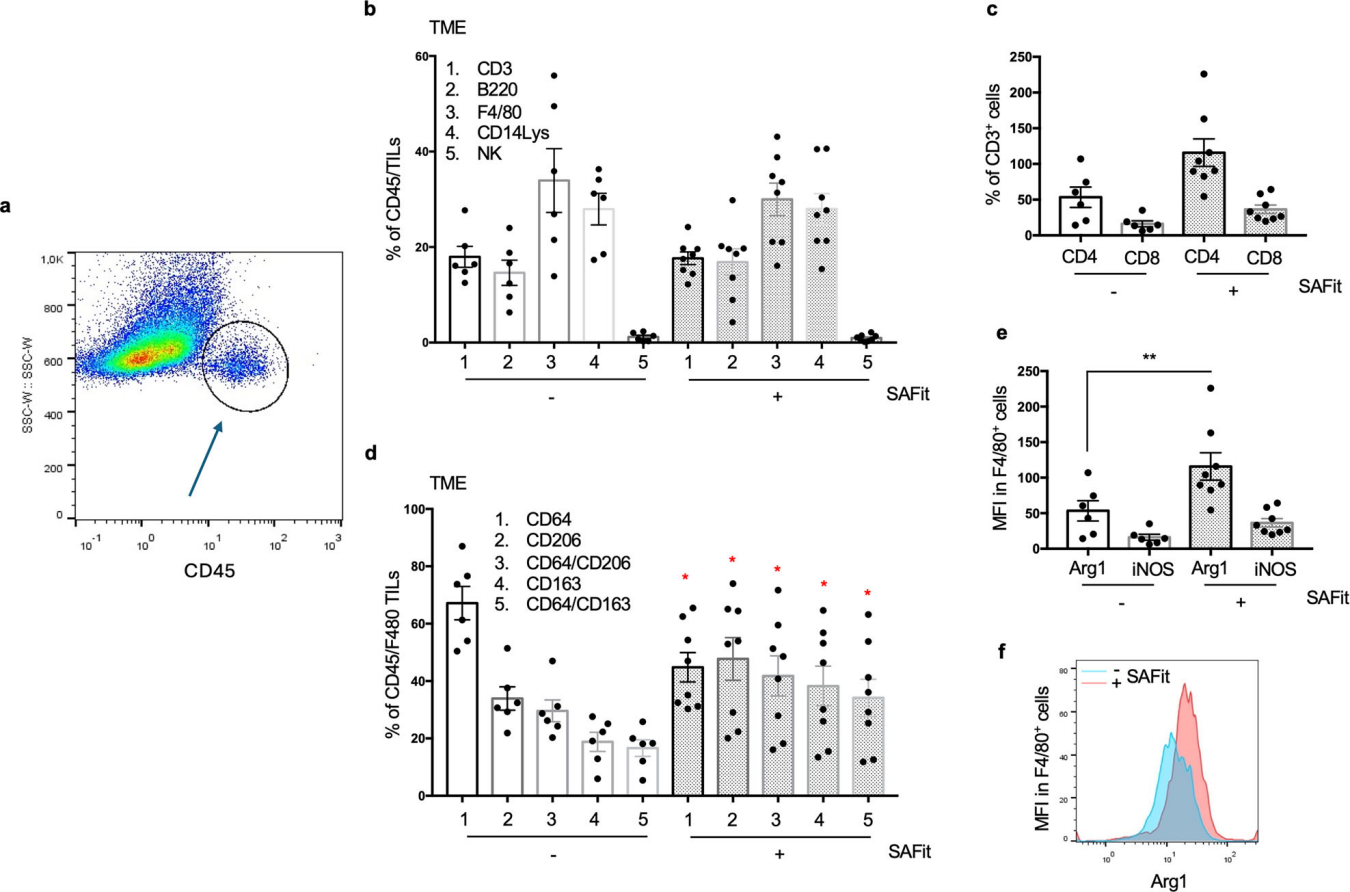
SAFit-induced changes of TME composition in TAMs. **a** Representative flow cytometry gating of CD45^+^ cells infiltrating the tumors. **b**, **c** Graphic representation of cell count values from TME immunophenotyping (Tumor Infiltrating Leukocytes TILs: macrophages, B and T cells, NK) of SAFit untreated (white histograms) or treated (grey histograms) tumors. **d** Characterization of F4/80 macrophage component of the TME. Graphical representation of counts of CD64 (M1 marker), CD163 and CD206 (M2 markers) and of CD64 co-expressing CD206 and CD163. **e** Arginase and iNOS expression (MFI) in F4/80^+^ cells infiltrating the tumors. **f** Representative flow cytometry histograms of Arginase expression are shown in overlay.

**Fig. 6:**
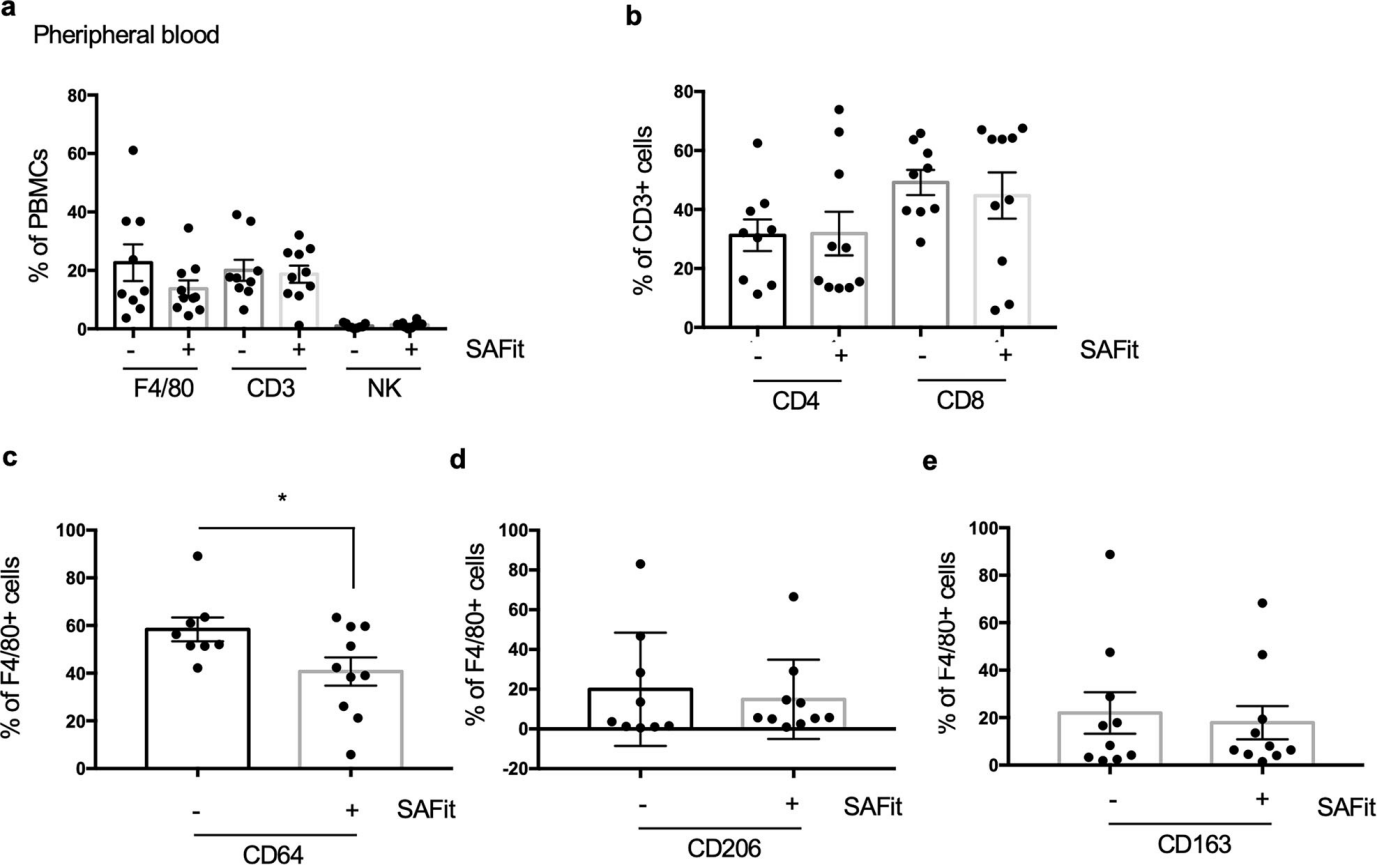
Immunophenotyping of PBMCs from SAFit treated and untreated mice. **a-e** Graphic representation of values from peripheral blood of untreated and SAFit-treated mice: F4/80, CD3 and NK counts (**a**); CD4 and CD8 counts (**b**); CD64 (**c**); CD206 (**d**); and CD163 counts (**e**).

Immunogenic cell death is fundamentally important in regulating the progression of melanoma tumors. Its pivotal role against this aggressive form of skin cancer is highlighted by dramatic results obtained with therapies that enhance the immune response. Macrophages are crucial cells in the innate immune response against tumors. As antigen-presenting cells, they enhance the activity of the adaptive immune system, particularly by stimulating effector T cells. Tumor-associated macrophages (TAMs) have a dual role, involving two competing interactions: they can reduce tumor growth through pro-inflammatory M1 polarization, or promote tumor growth through anti-inflammatory M2 polarization. We investigated the effect of SAFit on immune infiltrate of the tumor microenvironment (TME). TME was studied in flow cytometry using specific markers for Tumor Infiltrating Leukocytes (TILs). These markers included: F4/80, CD14Lys, CD64, CD163 and CD206 for monocytes/macrophages; CD3, CD4 and CD8 for T lymphocytes, B220 for B lymphocytes and NK (Fig. 5). CD45 served to gating immune cells (Fig. 5a). We could not find significant difference in composition of infiltrating T and B lymphocytes, F4/80 and CD14 macrophages, and NK between treated- and untreated-mice (Fig. 5b). Also, infiltration of CD4 and CD8 T cell subsets did not appear to be modified by SAFit (Fig. 5c). However, a subanalysis of F4/80 component revealed an M1 to M2 switch in TME of SAFit treated tumors (Fig. 5d). Measure of CD64 (M1 marker), CD206 and CD163 (M2 markers) revealed that the values (mean %±StDev) in untreated tumors (67.18% ± 14.25; 33.9 ± 9.98; 18.82 ± 8.13, respectively) were significantly different from those of SAFit-treated tumors (44.8 ± 14.5; 47.73 ± 21.02; 38.26 ± 19.58, respectively), indicating an increase of M2 profiles at the expenses of the M1 phenotype. CD64 were in total decreased by SAFit, moreover, the proportion of CD64 co-expressing CD206 and CD163 was increased in SAFit treated tumors (from 29.62 ± 9.29; 16.61 ± 7.03 to 41.66 ± 19.84; 34.16 ± 18.33, respectively). M1 to M2 switch was confirmed by the significant increase of Arginase expression in F4/80+ cells infiltrating SAFit-treated tumors (Fig. 5e,f). Supplementary information, Fig S1 shows representative flow cytometry histograms of F4/80 gating and measure of CD64, CD206 and CD163. In parallel, PBMCs were immunophenotyped (Fig. 6). F4/80, CD3 and NK counts were 22.63 ± 18.9; 20.02 ± 10.87; 0.94 ± 0.77; respectively, not different from those of SAFit-treated mice that were 13.63 ± 8.91; 18.72 ± 9.29; 1.36 ± 0.97; respectively (Fig. 6a). Similarly, CD4 and CD8 counts were 31.24 ± 16.12; 49.17 ± 12.74, respectively, not different from those of SAFit-treated mice that were 31.85 ± 23.43; 44.61 ± 24.7, respectively (Fig. 6b). Characterization of F4/80^+^ subset revealed that SAFit-treated mice had a significant decrease (p*) in CD64 expression (40.7 ± 18.74) in comparison with untreated mice (58.38 ± 14.04) (Fig. 6c). No difference was registered for CD206 (Fig. 6d) and CD163 (Fig. 6e) 19.96 ± 28.49; 21.98 ± 27.59 in untreated and 14.87 ± 19.96; 17.77 ± 22.08 in SAFit-treated. The expression levels of CD64, CD163 and CD206 in bone marrows showed no differences between the two groups (Supplementary information Fig. S2).

#### SAFit hampers the cytotoxic potential of CD8 TILs

**Fig. 7:**
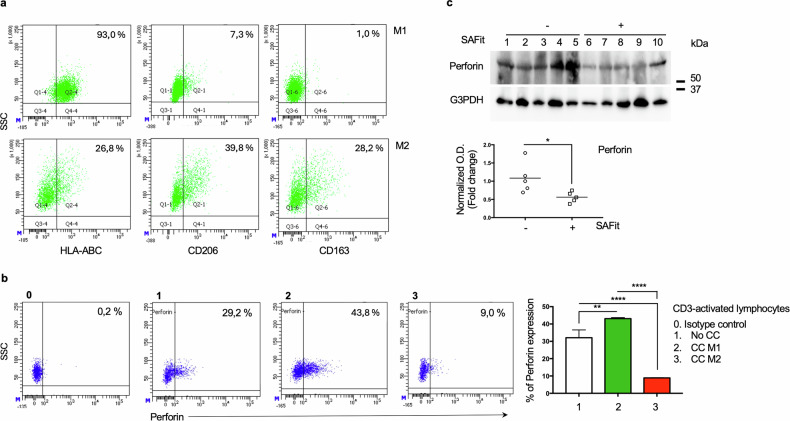
Levels of cytotoxic proteins are reduced by M2 macrophages and in SAFit-treated TMEs. **a** Representative flow cytometry histogram of polarized M1 or M2 stained with anti-HLA class I (M1 marker), CD206 and CD163 (M2 markers). **b** flow cytometry levels of perforin in cocultured autologous CD8 T lymphocytes. Compared with not cocultured lymphocytes (1), CD8 T lymphocytes cocultured with M1 (2) or M2 macrophages (3) showed increased and decreased perforin levels, respectively. **c** Western blot assay of perforin levels in lysates from tumor samples. G3PDH (analyzed on a blot run in parallel) was used as loading control. Full length western blots are shown as Supplemental Material. A densitometric analysis of bands was performed using ImageJ 1.42q for Macintosh.

The enrichment of the melanoma environment in M2 TAMs can promote the defective cytotoxicity of T-cells [15]. We performed an ex-vivo experiment of coculture of human primary M1 or M2 macrophages with autologous T lymphocytes to see whether the differently polarized macrophages could regulate the cytotoxicity of CD8 T lymphocytes in an opposite way. In vitro polarization of macrophages was assessed by flow cytometry (Fig. 7a). As expected, the phenotype of M1 exhibited higher levels of MHC class1 antigens and lower levels of CD206 and CD163 than M2 macrophages (Fig. 7a). Cytotoxicity capacity of CD8 T cells was evaluated through measure of perforin levels. Compared with not cocultured lymphocytes, CD8 T lymphocytes cocultured with M1 or M2 macrophages (Fig. 7b) showed increased and decreased perforin levels, respectively. A semiquantitative measure of perforin levels by Western blot assay of protein extracted by tumor samples showed reduced levels in SAFit-treated tumors (Fig. 7c). This result supports the hypothesis that SAFit-TME contained exhausted killer cells.

## Discussion

The present study provides in vitro elements in support of the efficacy of SAFit in turning off pro-tumoral pathways of the melanoma cell. FKBP51 inhibition by SAFit, indeed, sensitized melanoma cells to doxorubicin cytotoxicity, while ectopic expression of FKBP51 reduced the cooperative effect between doxorubicin and SAFit in accordance with the target selectivity of the small molecule. An EMSA showed that SAFit was effective in counteracting NF-κB activation, as previously suggested [4]. Accordingly, the expression of anti-apoptotic genes, which are targets of NF-κB transcription factors, resulted downmodulated by the small molecules. More precisely, mRNA and protein levels of *BCL-2* and *XIAP* were reduced in the presence of SAFit, suggesting that, by counteracting NF-κB activation, SAFit is able to turn off the survival pathways of the tumor. Melanoma cells treated with SAFit expressed reduced levels of TGF-β, its receptor signaling and capability of melanoma cells to migrate through transwell filters and invade the matrigel. These results are in line with previous studies conducted with melanoma xenografts in nude mice demonstrating the efficacy of FKBP51 siRNA in enhancing melanoma sensitivity to ionizing radiation and metastasis prevention [6, 7].

Surprisingly, the present study conducted in syngeneic mice with an intact immune system shows that intratumoral administration of SAFit did not contrast melanoma growth. Study of TME showed that SAFit-treated tumors developed M2-like profiles at the expenses of the M1 phenotype. At the same time, we observed that SAFit determined defective cytotoxicity of CD8 Tils suggesting that SAFit-TME contained exhausted killer cells. These findings suggest that the drug contributes to a rapid formation of a pro-tumoral TME. SAFit is a powerful inhibitor of NF-κB transcription factors [4], essential players in M1 pro-inflammatory action, which may explain, at least in part, the switch from M1 to M2 observed in SAFit treated TME. As widely recognized, M2 macrophages support cancer cells through various mechanisms; they produce multiple mediators that support the cancer stem cells, as well as several proteases that are involved in extracellular matrix remodeling and are critical sources of angiogenic factors [16]. Thus, the beneficial anti-melanoma effects that SAFit has in vitro demonstrated can be virtually reversed in vivo by the tumor-supporting activity of M2 macrophages [17]. Additionally, these TAMs act as drivers of immunosuppression within the TME, contributing to T-cell exhaustion [16]. Reduced perforin levels in SAFit-treated tumors are consistent with an exhausted phenotype. Our results underscore the pivotal role of the immune system in controlling melanoma growth. Combining SAFit with inhibiting agents of macrophage recruitment and re-educating macrophage polarization could result in an effective anti-melanoma strategy. This hypothesis deserves to be investigated in the future.

A key limitation of our study is that, despite our findings suggesting that SAFit treatment fosters an immune-tolerant and exhausted TME in melanoma, the current murine model leaves open the question of whether additional factors independent of the immune system influence the failure of the drug in vivo. Further research using a murine model that is not immunocompetent could help to understand better the mechanisms involved in SAFit’s lack of therapeutic effect in melanoma.

Another limitation is the lack of analysis of isolated tumor infiltrating CD45^+^ cells versus whole tumor that could provide deeper insights into how the infiltrate may impact on the results of RNA and protein assays.

## Materials and methods

### Cell culture, transfection and reagents

Human melanoma cell line SAN was obtained and cultured as described previously [6]. Human melanoma cell line A375 was kindly provided by CEINGE cell bank (Cellular Technology Platform, https://www.ceinge.unina.it/en/cell-cultures) at the Advanced Biotechnology Institute (Naples, Italy). Cell line A375 [18] was cultured in Dulbecco’s Modified Eagle’s Medium (DMEM) (Corning, Glendale, Arizona, USA) supplemented with 15% heat-inactivated fetal bovine serum (FBS; Corning), 200 mM glutamine (Corning), and 100 U/ml penicillin-streptomycin (Corning). Murine melanoma B16F10 cell line purchased by ATCC (ATCC-CRL-6475; Manassas, Virginia, USA). B16F10 were cultured in DMEM (Corning) with 10% heat-inactivated FBS (Corning), 200 mM glutamine (Corning), and 100 U/ml penicillin-streptomycin (Corning). Each cell line was tested for Mycoplasma after every thawing using a PCR-based method suitable for the detection of 11 mollicutes and capable of detecting all Mycoplasma species as indicated by Molla Kazemiha and colleagues [19]. After thawing, the cells were used in a range of passage numbers from the 4th to the 10^th^-12^th^ to keep safe the cell line identity. To obtain FKBP51-overexpressing SAN melanoma cells (Flag-FKBP51), the pcDNA3.1 + /C-(K)-DYK-tagged human FKBP51-transcript variant 1 expression vector (GenScript, Piscataway, New Jersey, USA) was transfected using Metafectene (Biontex, Munich, Germany), according to the manufacturer’s instructions. An empty vector was transfected to generate control cells (EV). Doxorubicin hydrochloride (Doxo), dacarbazine (DTIC) and rapamycin (RAPA) were from (Merck, Darmstadt, Germany) and were used at the concentration of 3, 27 μM and 100 nM, respectively. SAFit 1 and 2 were produced by Felix Hausch [11] and were used as described in specific sections.

#### Analysis of cell death

Analysis of DNA content by propidium iodide incorporation was performed in permeabilized cells by flow cytometry. Cells (2 × 10^4^) were harvested 48 h after the addition of doxorubicin or dacarbazine (DTIC), SAFit1 or SAFit2, washed in PBS, and resuspended in 150 μl of a solution containing 0.1% sodium citrate w/v, 0.1% Triton X-100 v/v, and 50 μg/ml propidium iodide (Sigma). After incubation at 4 °C for 30 min in the dark, cell nuclei were analyzed with a FACScan flow cytometer. Cellular debris was excluded from the analysis by raising the forward scatter threshold, and the DNA content of the nuclei was registered on a logarithmic scale. The percentage of the elements in the hypodiploid region was calculated.

#### Western blot assay

To obtain whole lysates, cells were collected, and cellular pellets were homogenized in modified RIPA buffer (50 mM Tris-HCl pH 7.5, 125 mM NaCl, 1% NP-40, 0.25% Na-deoxycholate, 1 mM Na-fluoride, 1 mM Na-orthovanadate, 1 mM phenylmethanesulfonylfluoride (PMSF), 1 mM dithiothreitol (DTT), protease inhibitor cocktail) [20]. After 30 min of incubation on ice, lysates were centrifuged ad 14.000 rpm for 15 min to remove cell debris and the supernatant was saved for Western blot assay. Protein concentration was determined using the Bradford protein assay (Bio-rad, Hercules, CA, USA), measuring absorbance at 595 nm. Cell lysates were equalized for total proteins, and the final volume was levelled with water and Laemmli sample buffer (LB). Samples were denatured for 5 min at 95 °C, then loaded in 8/10% T SDS-PAGE and transferred onto a methanol-activated PVDF membrane (Immobilon-P, Millipore, Merck). The membranes were incubated with the corresponding primary antibody, at 4 °C, overnight. Primary antibodies against the following proteins were diluted as follows: Flag (M2, mouse monoclonal, Merck) 1:5000; β-Actin, (15G5A11/E2, mouse monoclonal, Thermo Fisher Scientific, Waltham, Massachusetts, USA) 1:5000; TGF-β (V; rabbit polyclonal, Santa Cruz Biotechnology) 1:500; γ-Tubulin (GTU-88, mouse monoclonal, Merck) 1:5000; phospho-Smad2 (Ser465/467, Cell Signaling Technology, Danvers, MA, USA) 1:500; Smad 2/3 (H465, rabbit polyclonal, Santa Cruz Biotechnology, CA, USA) 1:500; TGFβ RI (V-22, rabbit polyclonal, Santa Cruz Biotechnology), PD-L1 (NBP1-76769, Novus Biological) 1:1000; anti-Caspase 3 **(**clone 84803.111, Mouse Monoclonal Sigma); G3PDH (6C5, mouse monoclonal, Merck) 1:5000; Perforin PE-CF594 (Clone δG9, Invitrogen-Thermo Fisher Scientific) 1:500. Bcl-2 (N-19, rabbit polyclonal, Santa Cruz Biotechnology), XIAP (2F1, mouse monoclonal, Stressgen Biotechnologies, BC, Canada), FKBP51 (NB100-68240, Novus Biological), Cyclin D1 (92G2, rabbit polyclonal, Cell Signaling Technology). After washes, membranes were incubated with secondary antibodies for 1 h, at room temperature. Anti- mouse and anti-rabbit secondary antibodies HRP-conjugated were purchased from ImmunoReagents (Raleigh, Carolina del Nord, USA) and diluted 1:5000. IBs were revealed with Western Blotting Luminol Reagent (Santa Cruz Biotechnology). A quantification of bands was obtained by densitometry analysis using ImageJ 1.42q for Macintosh; integrated optical densities (ODs) of each analyzed protein were normalized to a relative housekeeping gene, and values were expressed as fold change of protein levels in the different samples in comparison with a control sample whose expression was arbitrarily indicated equal to 1 [21]. Full length western blots are uploaded as Supplemental Material.

#### Electrophoretic mobility-shift assay (EMSA)

SAN melanoma cells were cultured with 3 μM doxorubicin and SAFit1 (20 nM) or SAFit2 (30 nM) for 3 and 5 h. Cell nuclear extracts were prepared from 1×10^6^ cells, stimulated as described, by homogenization of the pellet in two volumes of 10 mM HEPES, pH 7.9, 10 mM KCl, 1.5 mM MgCl_2_, 200 mM EDTA, 0.5 mM DTT, 0.5 mM PMSF, and 10% glycerol (v/v). Nuclei were centrifuged at 1000xg for 5 min, washed, and resuspended in two volumes of the above-specified solution. KCl (3 M) was added until the concentration reached 0.39 M. Nuclei were extracted at 41 °C for 1 h and centrifuged at 10000×g for 30 min. The supernatants were clarified by centrifugation and stored at −80 °C. Protein concentrations were determined using the Bradford method. The NF-κB consensus 5’-CAACGGCAGGGGAATCTCCCTCTCCTT-3’ oligonucleotide was end-labeled with [γ^32^P] ATP using a polynucleotide kinase (Roche, Basel, Switzerland). End-labeled DNA fragments were incubated at room temperature for 20 min with 5 μg of nuclear protein, in the presence of 1 μg poly(dI-dC), in 20 ml of a buffer consisting of 10 mM Tris-HCl, pH 7.5, 50 mM NaCl, 1 mM EDTA, 1 mM DTT, and 5% glycerol (v/v). In competition assays, a 50x molar excess of NF-κB or NFAT cold oligo was added to the incubation mixture. Protein-DNA complexes were separated from the free probe on a 6% polyacrylamide (w/v) gel run in 0.25x Tris borate buffer at 200 mV for 3 h at room temperature. Gels were dried and exposed to X-ray film (Fuji film, L.E.P., Naples, Italy).

#### qPCR

Total RNA was extracted from cells by TRIzol (Thermo Fisher Scientific). Each RNA was used for cDNA synthesis with iScript Reverse Transcription (Bio-Rad, Hercules, California, USA). Relative gene expression was quantified by qPCR with 2^-DDCt^ comparative method using the SsoAdvancedTM SYBR Green Supermix (Bio-Rad) and specific qPCR primers. Oligo primers used for *XIAP*, *TβRI*, *FKBP51* were purchased from Qiagen (validated QuantiTect primers, San Diego, California, USA) and run along with coamplified housekeeping genes β-Actin and *18S* whose sequences were previously reported [4]. Other oligo sequences are reported: hBCL-2-Fw: 5’-CTGCACCTGACGCCCTTCACC-3’, hBCL-2-Rev: 5’-CACATGACCCCACCGAACTCAAAGA-3’; m-*CCND1*, m-*TGF-β*, m-*PDL-1* and m-*HRP* sequences are reported in [22–24].

#### Invasion assay

The invasion assay was performed in accordance with Albini and Benelli [25]. Briefly, this method is a simple modification of Boyden chamber assay because of matrigel addition, which mimics the physiological barrier to tumor cells, allowing to evaluate both migratory and invasive capabilities of melanoma cells. Transwell chambers, with 8 mm polyester filters, were used for this assay (Corning Incorporated Life Sciences, Tewksbury, MA, USA) and coated with 100 μl of basement membrane matrix, matrigel (Becton Dickinson, Franklin Lakes, NJ, USA). The matrigel was diluted with cold PBS 1x, applied to the filters, dried in humidified atmosphere at 37 °C for at least 5 h and reconstituted with serum-free medium. The coated filters were placed in Boyden chambers, forming two medium-filled compartments separated by such microporous membrane. A nutrient gradient was created between the two chambers (1% FCS medium in the upper and 10% FCS medium in the lower compartment). A375 melanoma cells (75×10^3^) were cultured for 2 days in the presence of SAFit1 (20 nM) or SAFit2 (30 nM); then, cells were placed in the upper compartment and, after a 24 h incubation time, the membrane between the two compartments was fixed and stained with 0.25% Crystal Violet in methanol. Cells at the lower surface of the filter were photographed and counted; then, the Crystal Violet was eluted in 1% SDS and read in a spectrophotometer at a wavelength of 570 nm.

#### Animal studies, housing and treatment

C57BL/6 mice were purchased from Envigo (Telangana, India) and were housed at the Federico II University DMMBM animal facility under controlled temperature (22 ± 2 °C), humidity (55 ± 5%), light-controlled conditions (12 h light:12 h dark cycle), ad libitum access to food and water and pathogen-free conditions. All animal care procedures and experiments were approved by the Institutional Animal Care and Use Committee (Autorization n° 253/2020-PR). All efforts were made to minimize animal suffering during the experiment. Briefly, at the time of mice injection, B16 cells were added with SAFit2 at the concentration of 20 mg/kg or DMSO. Then, 5×10^5^ cells were injected subcutaneously on the right hind flank in 100 μL PBS1x. Ten mice were injected with SAFit2-treated cells and 10 mice with DMSO-treated cells. Tumors were monitored each couple of day to avoid exceeding the maximal tumor size/burden allowed by the ethics committe. Before sacrifices, mice were anesthetized with Tiletamine-Zolazepam 30 mg/kg via intraperitoneal puncture to minimize stress and discomfort. At the scheduled sacrifice time (20 days from the injection), mice were sacrificed by CO_2_ asphyxiation and peripheral blood samples were collected from the maxillary venous sinus using a sterile lancet. One mouse injected with DMSO-treated cells died before the sacrifice. A total of 300 µL of peripheral blood per animal was collected in EDTA tubes, with 50 µL used for each immunofluorescence (IF) as described in the *“Flow cytometry”* section. Tumors were harvested from mice and after immersion in ice-cold 10% FBS RPMI-1640 medium were mechanically dissociated using sterile surgical instruments. The tissue fragments were further disaggregated into single-cell suspensions by manual processing through a 70 µm cell strainer. Dissociated cells, containing both tumor and immune infiltrates, were subjected to differential centrifugation using a Ficoll-Hypaque density gradient (Histopaque-1077, Merck) to isolate murine tumor infiltrating leukocytes (TILs), that were then processed for IF, as described in the *“Flow cytometry”* section.

#### Peripheral blood mononuclear cells studies

Human peripheral blood mononuclear cells (PBMCs) were separated by differential centrifugation using a Ficoll-Hypaque density gradient (Histopaque-1077, Merck), washed, and resuspended in RPMI1640 medium (Corning) supplemented with 10% heat-inactivated fetal bovine serum (FBS; Corning), 200 mM glutamine (Corning), and 100 U/ml penicillin-streptomycin (Corning). To differentiate human macrophages, PBMCs were stimulated for 6 days with M-CSF (Immunotools, Friesoythe, Germany) used at a final concentration of 50 ng/mL and then differentiated into M1 macrophages with 100 ng/mL IFN-γ (Immunotools) and 100 ng/mL LPS (Merck) or M2 macrophages with 50 ng/mL IL-4 (Immunotools) for 48 h [26]. PBMC activation was conducted by stimulating cells with 10 ug (per 1 × 10^6^ cell) CD3 Monoclonal Antibody (OKT3), Functional Grade (Thermo Fisher Scientific) for 16 h. After activation, PBMCs were co-cultured with autologous monocyte-derived M1 or M2 macrophages for 24 h. Following co-culture, both macrophages and activated PBMCs were harvested and processed for immunofluorescence staining, as described in the *“Flow cytometry”* section.

#### Flow cytometry

Human PBMCs (activated lymphocytes and differentiated macrophages), murine TILs and murine whole blood were stained to be assayed by flow cytometry as follows. Five-10 μl of antibody recognizing the typical cluster differentiation (CD) were added to 50 μl of whole blood, 1×10^6^ PBMCs or TILs and incubated for 30 min in the dark at 4 °C. For mice IF, the following antibodies were used: anti-mouse-F4/80-PE-Vio770 (Clone REA126, Miltenyi Biotec, Gladbach, NWR, Germany), anti-mouse-CD45-VioGreen (Clone REA737, Miltenyi Biotec), anti-mouse-CD45R/B220 FITC (Clone RA3-6B2, Biolegend, San Diego, California, U.S), anti-mouse-CD68-VioBlue (Clone REA835, Miltenyi Biotec), anti-mouse-CD64-APC-Vio770 (Clone REA286, Miltenyi Biotec), anti-mouse-CD163 PE (Clone TNKUPJ, Thermo Fisher Scientific) anti-mouse-CD206 FITC (Clone MR5D3, Thermo Fisher Scientific), anti-mouse-CD3-VioBright B515 (Clone REA641, Miltenyi Biotec), anti-mouse-CD8a-PE-Vio770 (Clone REA601, Miltenyi Biotec), anti-mouse-CD14-APC-Vio770 (Clone REA934, Miltenyi Biotec), anti-mouse-NK1.1-APC (Clone REA1162, Miltenyi Biotec), anti-mouse-Ly-6G-VioBlue (Clone REA526, Miltenyi Biotec), and anti-mouse-MHC Class II-PE (Clone REA813, Miltenyi Biotec). For mice whole blood samples, red blood cell lysis was performed after antibody incubation by adding 450 μL of 1X FACS Lysing Solution (BD Pharmingen, San Jose, CA, USA), followed by gentle vortexing and incubation for 15 minutes in the dark at room temperature. Co-cultured human macrophages were stained with anti-human-HLA-ABC FITC (Clone W6/32, Immunotools), anti-human-CD206 BV480 (Clone 19.2, BD Pharmingen), anti-human-CD163 PE-CF594 (Clone GHI/6, BD Pharmingen). Activated human lymphocytes were stained with anti-human-CD3 BV510 (Clone HIT3a, BD Pharmingen) and anti-human-CD8 APC (Clone UCHT-4, Immunotools). All samples were washed with Flow Cytometry Staining Buffer before further processing. For intracellular staining, 200 μL of a fixation/permeabilization buffer (BD-Pharmingen Cytofix/Cytoperm Kit) was added to each tube. Samples were incubated for 20 min in the dark at 4°C. Murine cells were stained with anti-Arginase 1 Alexa Fluor 488 (Clone AlexF5, Thermo Fisher Scientific) and anti-iNOS PE (Clone REA982, Miltenyi Biotec). Activated human lymphocytes were stained with anti-human-Perforin PE-CF594 (Clone δG9, BD-Pharmingen). Isotype-conjugated control antibodies were used to assess nonspecific binding. Flow cytometry acquisition was performed using a MACSQuant Analyzer 10 flow cytometer (Miltenyi Biotec) or a BD Facs Celesta (BD Pharmingen). All data were analyzed using FlowJo software.

#### Statistical analysis

The results reported are the mean and standard deviation of independent experiments. The statistical significance of differences between means was estimated using Student’s *t*-test. A *p*-value ≤ 0.05 was considered statistically significant. *P < 0.05, **P < 0.01, ***P < 0.001, ****P < 0.0001.

## Supplementary information

Supplementary_uncropped files

Supplementary figure S1 and S2

## Data Availability

All data generated or analysed during this study are included in this published article and its supplementary information files.
