# Supplementary figures and images for: Exploring the potential of selective FKBP51 inhibitors on melanoma: an investigation of their in vitro and in vivo effects
Source: Cell Death Discov. 2025 Apr 3;11:138. doi: 10.1038/s41420-025-02430-y (PMC11969000; doi:10.1038/s41420-025-02430-y)

Fig.1b

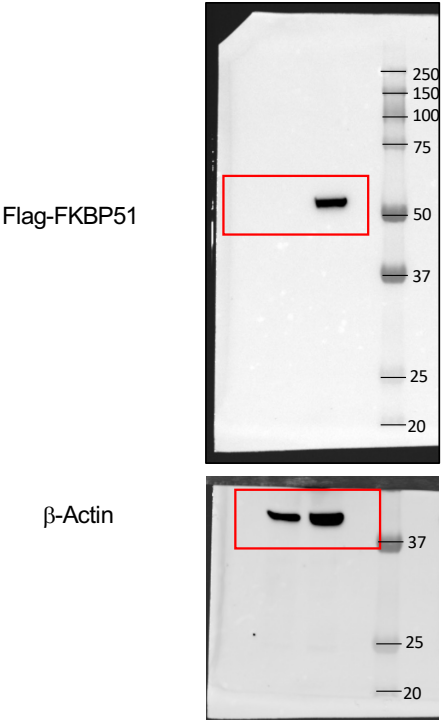

Fig.2a

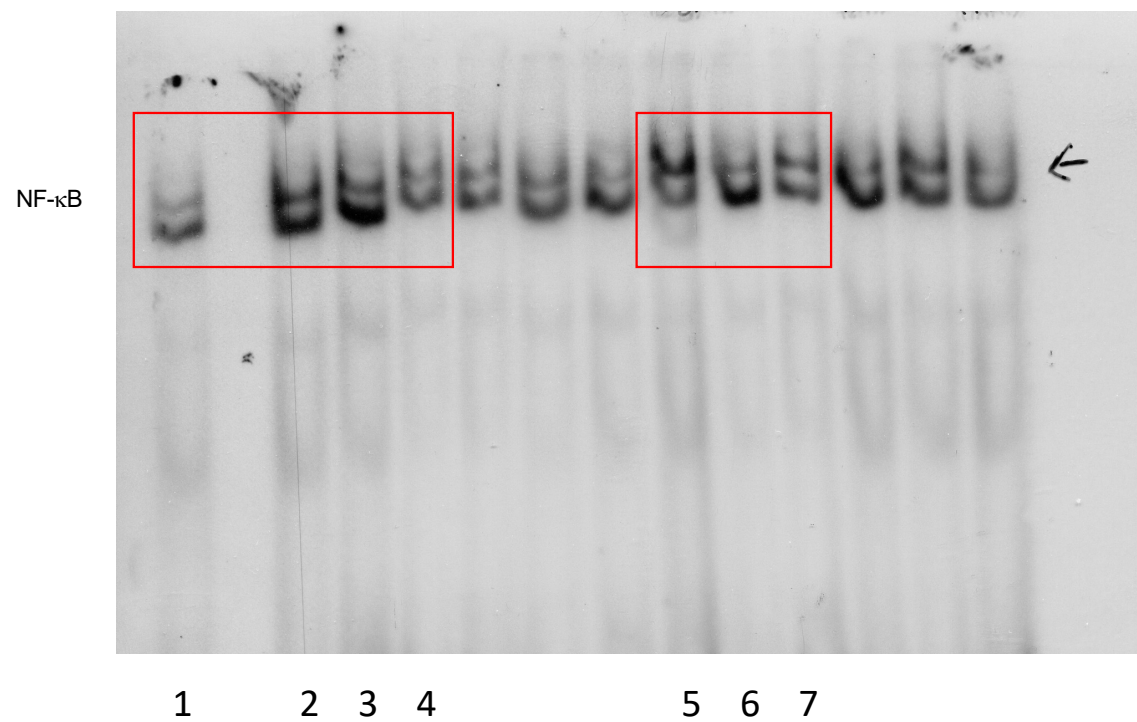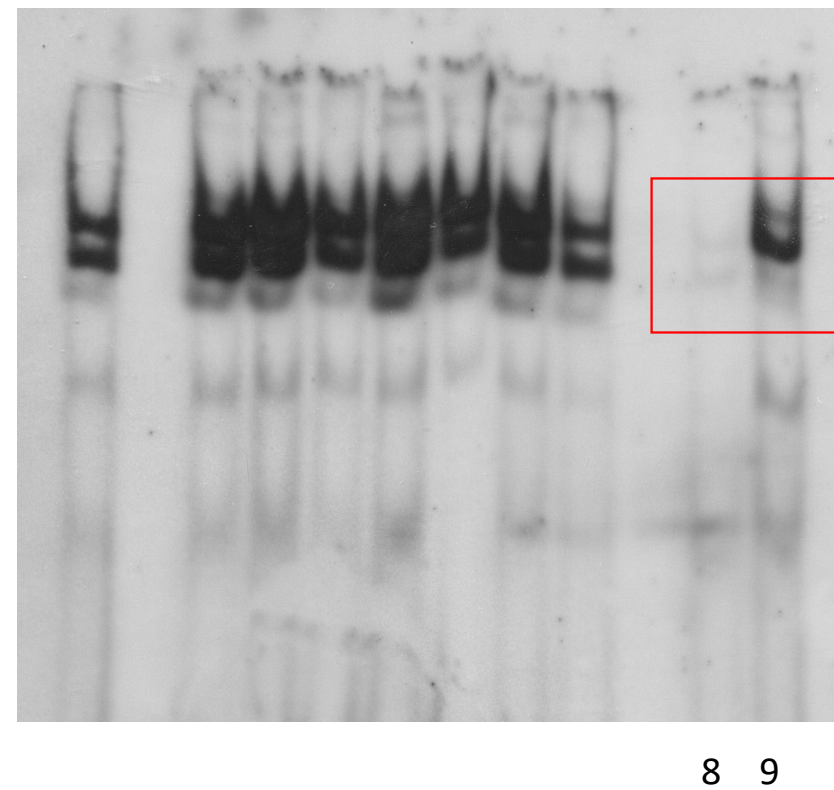

Fig.2b

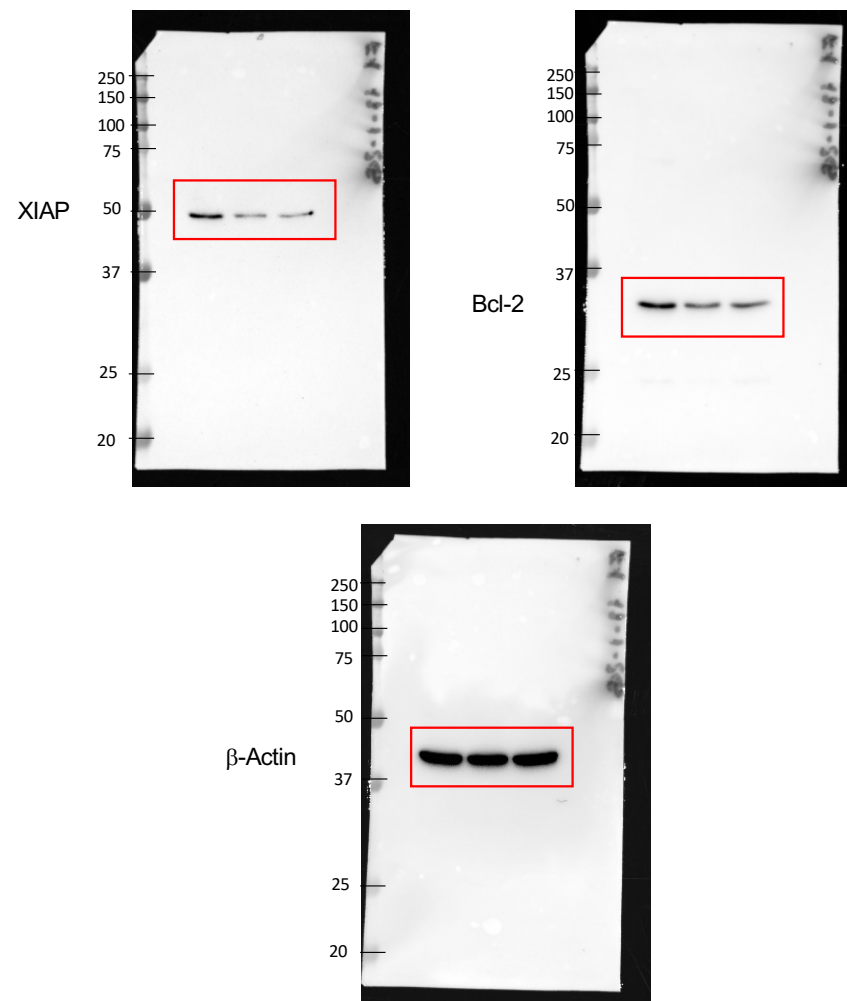

Fig.2c

Flag-FKBP51

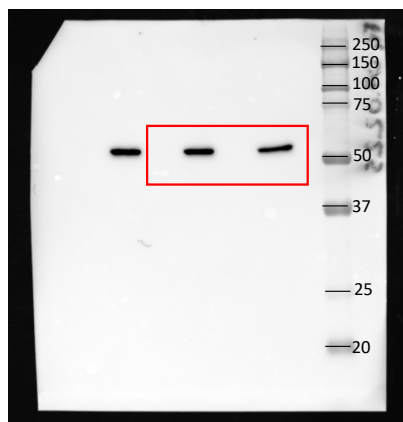

Bcl-2

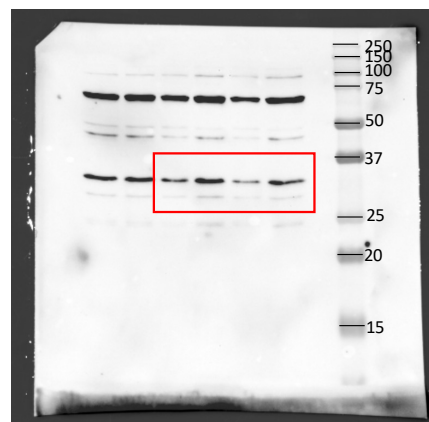

FKBP51

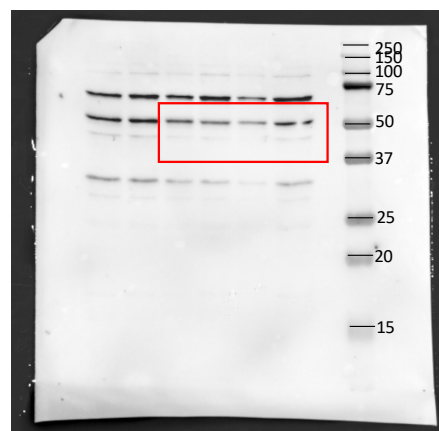

G3PDH

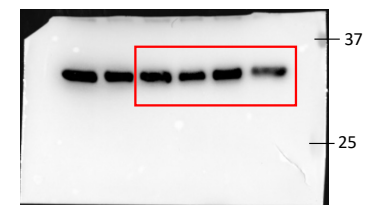

Fig 3a

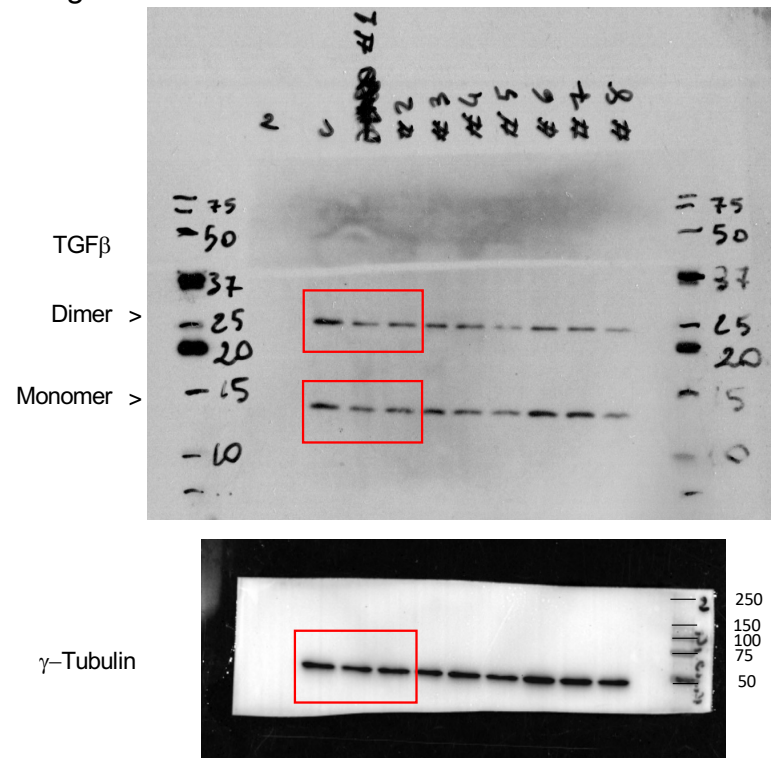

Fig 3b

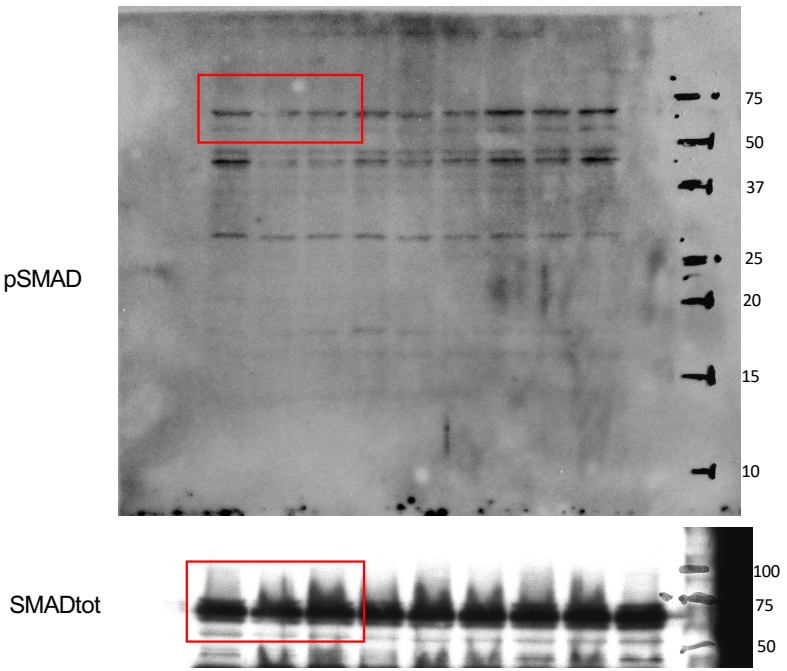

Fig 3c

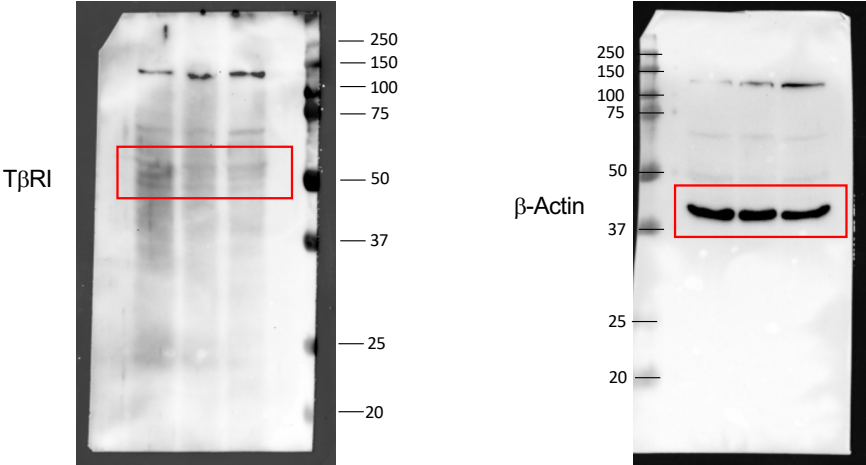

Fig 4b

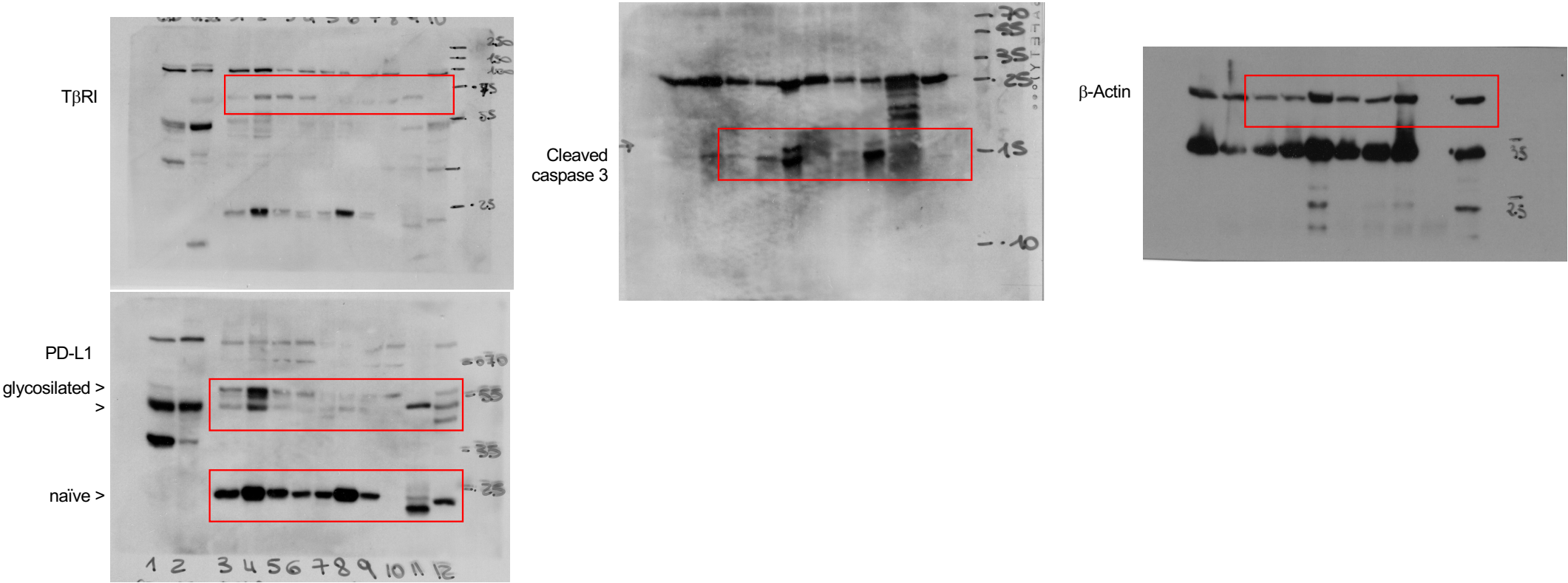

Fig 4c

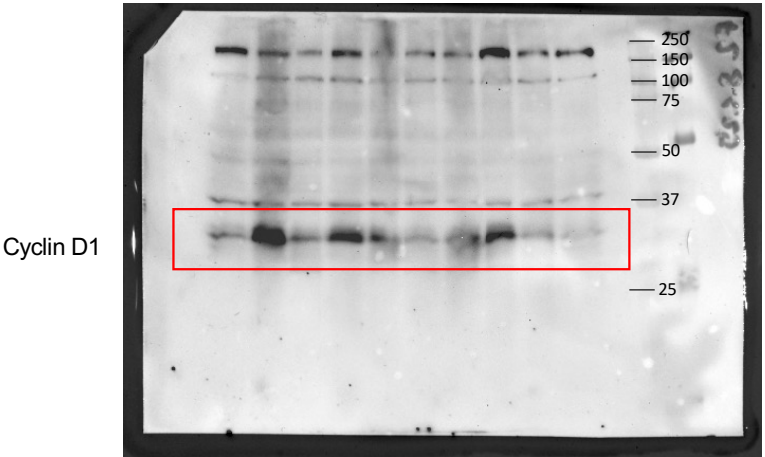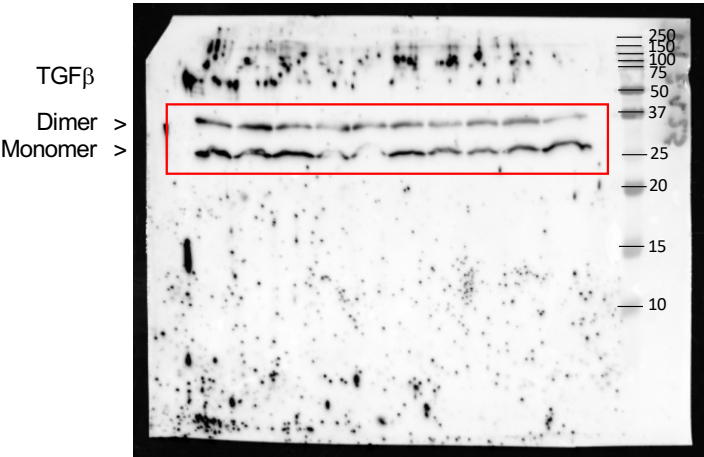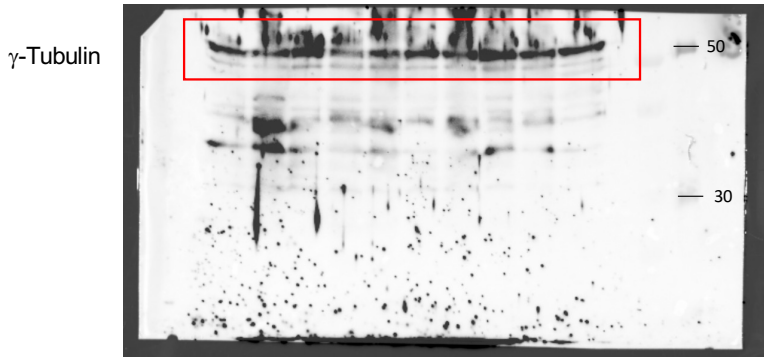

Fig 7c

Perforin

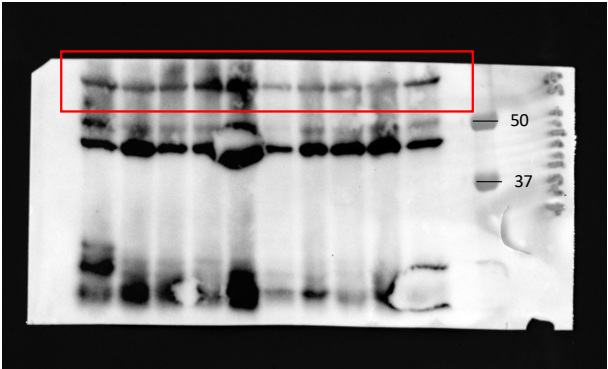

G3PDH

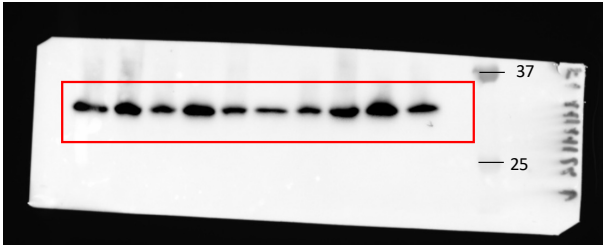

Supplement: Supplementary file 1 — Supplementary_uncropped files [file 41420_2025_2430_MOESM1_ESM.pdf]
