# Supplementary material for: Exploring the potential of selective FKBP51 inhibitors on melanoma: an investigation of their in vitro and in vivo effects
Source: Cell Death Discov. 2025 Apr 3;11:138. doi: 10.1038/s41420-025-02430-y (PMC11969000; doi:10.1038/s41420-025-02430-y)
Supplement: Supplementary file 2 — Supplementary figure S1 and S2 [file 41420_2025_2430_MOESM2_ESM.pdf]

Supplementary material

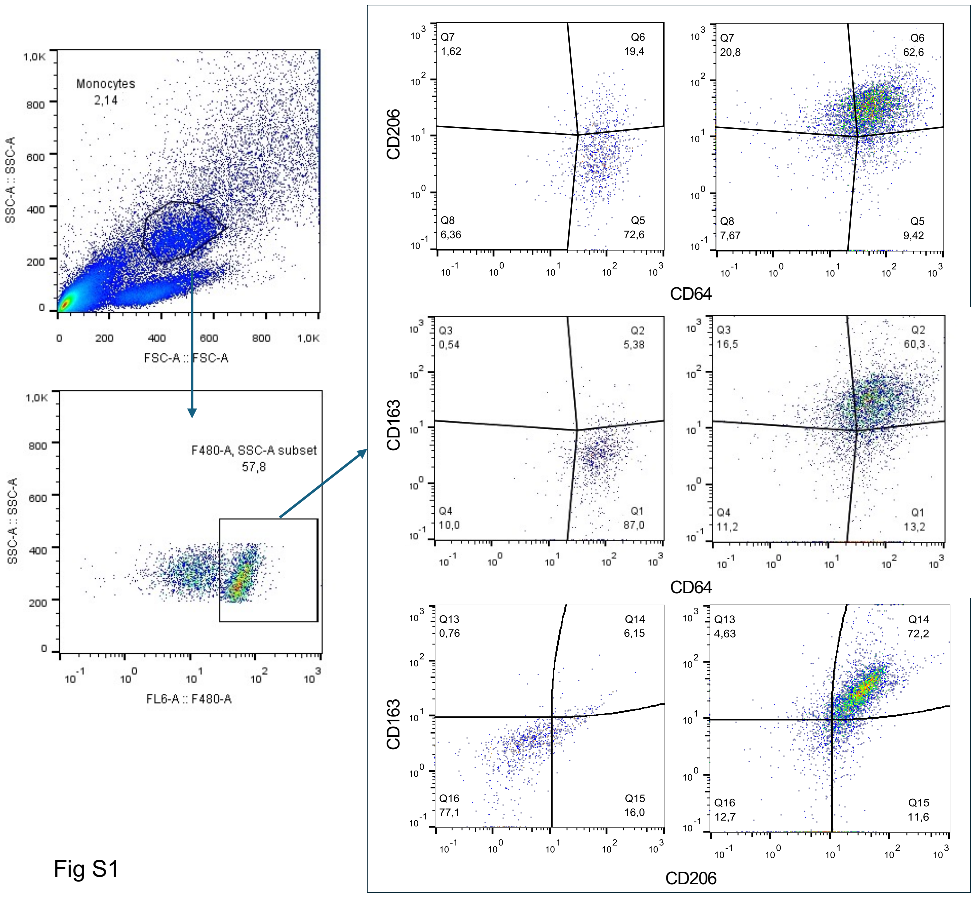

Figure S1 Representative flow cytometry histograms illustrating the gating strategy for monocytes/macrophages in tumor samples, with and without SAFit treatment. An initial gate was performed to identify the monocyte/macrophage population based on forward scatter (FSC) and side scatter (SSC) parameters. Then, a subsequent gate was applied to isolate the F4/80^{+} subset. Within this subset, the biparametric analysis of CD206/CD64, CD163/CD64, and CD163/CD206 subpopulation was performed as shown by dot plots.

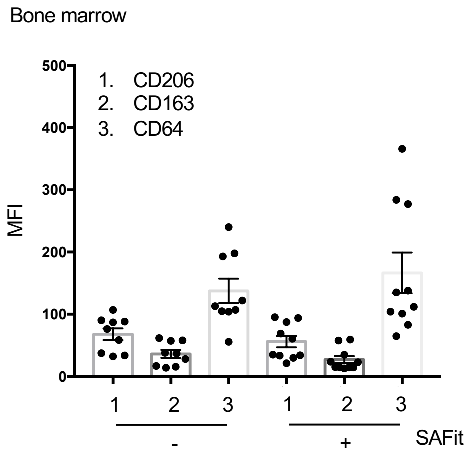

Fig S2

Figure S2 Graphical representation of mean values (columns) and standard deviations (bars) of the expression levels (mean fluorescence intensities, MFI) relative to CD206, CD163, and CD64 in bone marrow-derived macrophages (BMDMs) from mice treated with or without SAFit. Bone marrow was extracted from the tibia and femur after the removal of surrounding muscle. The bones were cut at the joints, and marrow was flushed out using a 25-gauge needle attached to a 10 mL syringe filled with DMEM. Red blood cells were lysed, and cell clumps were disaggregated using a needle-less syringe before passing the suspension through a 70 µm cell strainer. The resulting cells were cultured in DMEM supplemented with 20% L929-conditioned medium for 6 days, followed by immunofluorescence staining for flow cytometry analysis.
